# Platelet-neutrophil interaction in COVID-19 and vaccine-induced thrombotic thrombocytopenia

**DOI:** 10.3389/fimmu.2023.1186000

**Published:** 2023-05-19

**Authors:** Johannes Hirsch, Günalp Uzun, Jan Zlamal, Anurag Singh, Tamam Bakchoul

**Affiliations:** ^1^ Institute of Clinical and Experimental Transfusion Medicine, University Hospital of Tuebingen, Tuebingen, Germany; ^2^ Center for Clinical Transfusion Medicine, University Hospital of Tuebingen, Tuebingen, Germany

**Keywords:** COVID-19, platelet activation, coagulopathy, VITT, neutrophil extracellular traps (NET), immunothrombosis

## Abstract

Coronavirus disease 2019 (COVID-19) is known to commonly induce a thrombotic diathesis, particularly in severely affected individuals. So far, this COVID-19-associated coagulopathy (CAC) has been partially explained by hyperactivated platelets as well as by the prothrombotic effects of neutrophil extracellular traps (NETs) released from neutrophils. However, precise insight into the bidirectional relationship between platelets and neutrophils in the pathophysiology of CAC still lags behind. Vaccine-induced thrombotic thrombocytopenia (VITT) is a rare autoimmune disorder caused by auto-antibody formation in response to immunization with adenoviral vector vaccines. VITT is associated with life-threatening thromboembolic events and thus, high fatality rates. Our concept of the thrombophilia observed in VITT is relatively new, hence a better understanding could help in the management of such patients with the potential to also prevent VITT. In this review we aim to summarize the current knowledge on platelet-neutrophil interplay in COVID-19 and VITT.

## 1 Introduction

Coronavirus disease 2019 (COVID-19) is caused by infection with severe acute respiratory syndrome coronavirus 2 (SARS-CoV-2). The disease was initially recognized as a predominantly respiratory illness after its first appearance in the city of Wuhan, China in late 2019, but the presence of the virus at extrapulmonary sites and its fatal effects were subsequently demonstrated (1–3).

COVID-19 patients often suffer from coagulopathy in addition to mortality due to respiratory failure. These are mainly consequences of the prothrombotic state, especially in moderate and severe cases. Venous thromboembolism (VTE), thrombocytopenia and disseminated intravascular coagulation (DIC) were early described as common complications in SARS-CoV-2 infected patients (4–7). Agarwal et al. calculated the overall prevalence of VTE to be as high as 20.7%, with the risk being doubled in COVID-19 cases admitted to the intensive care unit (8). A large retrospective analysis of more than 370,000 cases from England found that 86% of hospitalized COVID-19 patients with VTE also suffered a concomitant pulmonary embolism, highlighting the coagulation-related risks in COVID-19 (9).

The pathophysiology of COVID-19-associated coagulopathy (CAC) is still under investigation and both cellular and plasmatic constituents of the coagulation system appear to be affected by infection with SARS-CoV-2. It has been established that hyperactivated platelets play a major role in CAC (10–14). Recently, the contributions of cells of the immune system during thrombus formation have been discussed in the setting of immunothrombosis. Histopathological examinations of thrombi from COVID-19 patients have demonstrated an increased deposition of neutrophils within the thrombus matrix in the lung vasculature suggesting that platelet-neutrophil interplay may be crucial in initiation and perpetuation of thrombosis in (hyper-)inflammatory diseases such as COVID-19 (13, 15–18). Several studies have already shown an increase in platelet-neutrophil aggregates (PNAs) circulating in blood of COVID-19 patients. However, the exact mechanisms of interaction between platelets and neutrophils remain unclear as little research was conducted yet on how these cells interact in promoting CAC. Next to their ability of phagocytosis and secretion of antimicrobial enzymes, neutrophils are capable of releasing neutrophil extracellular traps (NETs) mainly consisting of DNA (19). NETs serve as attachment structures for enzymes such as myeloperoxidase (MPO) or neutrophil elastase but also trap pathogens and allow their degradation by the antimicrobial substances. NETs have been studied extensively in the last decade for their impact on thrombus formation (20, 21). Various prothrombotic conditions including DIC in septic patients (22), neoplasms (23) and heparin-induced thrombocytopenia (HIT) (24, 25) have been found to be associated with elevated levels of NETs. SARS-CoV-2 was also demonstrated to directly induce NETosis (26–28). Thus, the detrimental consequences of severe COVID-19 have been partially attributed to both direct and indirect effects of NETs.

Up until now several vaccine candidates have been approved worldwide to mitigate the burden on society and healthcare caused by the COVID-19 pandemic. Among the first vaccine platforms authorized in Europe were mRNA-based vaccines and vaccines using adenoviral vectors. Shortly after the rollout of immunization programs, cases of thrombocytopenia accompanied by thrombotic events have been reported in individuals recently vaccinated with Vaxzevria (ChAdOx1 nCoV-19 vaccine, AstraZeneca) or Janssen Covid-19 vaccine (Ad26.COV2.S, Johnson & Johnson) – both vaccines relying on the adenoviral vector technique. For the first (or unknown) immunization with Vaxzevria, the UK´s Medicines and Healthcare products Regulatory Agency (MHRA) calculated the overall reported incidence of thromboembolic events associated with thrombocytopenia to be 15.9 cases/million doses (29). This specific syndrome, termed vaccine-induced thrombotic thrombocytopenia (VITT), is caused by the formation of antibodies against platelet factor 4 (PF4). Thrombosis and particularly cerebral venous sinus thrombosis (CVST) is the key finding of VITT with the case fatality rate estimated to be approximately 18% (30, 31). The diagnosis of VITT usually requires a history of immunization with an adenoviral vector anti-SARS-CoV-2 vaccine (mainly Vaxzevria or Janssen) minimum 4 days prior, detection of anti-PF4 antibodies in serum and additional more specific platelet aggregation tests (32–34). Treatment options in VITT include non-heparin anticoagulants, administration of intravenous immunoglobulins (IVIG) as well as supportive care (35, 36).

Here, we give an overview of the current state of research on the interaction between platelets and neutrophils in CAC and VITT.

## 2 Platelets and neutrophils in CAC

### 2.1 Altered platelet functionality in COVID-19

Besides a reduction in platelet count, the functional properties of platelets are reportedly deranged during COVID-19. Platelets of patients infected with SARS-CoV-2 were found to have higher expression of activation markers than non-COVID-19 controls (11, 12, 37–40). **Table 1** lists markers for platelet and neutrophil activation described in COVID-19. Moreover, platelets in COVID-19 had an increased tendency towards aggregation and showed greater responses to stimuli as ADP, thrombin receptor activator peptide 6 (TRAP-6) or thrombin itself (10, 14, 37, 38). This indicates both a hyper-active and hyper-reactive platelet phenotype during infection with SARS-CoV-2.

**Table 1:** Markers of platelet and neutrophil activation described in COVID-19.

	Found in/on	Clinical significance	Citations
Platelets			
P-Selectin/CD62P	α granules	parameter of platelet activation	(12, 13, 37–39, 41)
Phosphatidylserine (PS)	cell membrane	procoagulant platelets defined as PS^+^/CD62P^+^	(11, 12)
CD63	δ granules	parameter of platelet activation	(39)
PAC-1	activated GP IIb/IIIa	parameter of platelet activation	(13, 37)
Mitochondrial membrane potential, ΔΨm (e.g., TMRE)	mitochondria	loss of ΔΨm is seen in procoagulant and apoptotic platelets	(11)
Neutrophils			
CD66b	granulocyte membrane	general PMN marker, but also increases with activation/degranulation	(42, 43)
CD11b and CD18	Mac-1	markers of activation	(13, 38, 42–45)
Citrullinated histone (CitH3)	formed in process of NETosis, part of NETs	markers for NETosis	(40, 41, 46)
Myeloperoxidase (MPO) or MPO-DNA complexes	Primary (azurophilic) granules, part of NETs		

Direct and indirect aspects of platelet activation have been proposed. Zhu et al. confirmed the presence of SARS-CoV-2 RNA within platelets. Six out of the seven patients with this finding deceased shortly after. On the contrary, only one out of 24 COVID-19 patients from the survivor group was found to have RNA positive platelets (47). The principal mechanism of cellular uptake of SARS-CoV-2 is assumed to occur *via* the angiotensin-converting enzyme (ACE2) receptor in combination with the transmembrane serine protease/serine subfamily member 2 (TMPRSS2) (48). However, whether ACE2 and TMPRSS2 are expressed on platelets is still under debate and other mechanisms of viral entry have also been proposed (37, 49, 50). Furthermore, CD147 may serve as a site of direct interaction between platelets and SARS-CoV-2 and was also described to be a mediator of viral entry into cells *via* endocytosis (51, 52). Additionally, glycoprotein Ib (or CD42b) was identified as a receptor used by the spike protein of SARS-CoV-2 (53). Furthermore, the direct effects of SARS-CoV-2 on platelets appear to be mediated through the upregulation of both caspase-dependent (apoptosis) and caspase-independent pathways (necroptosis) (54, 55).

Examples for indirect mechanisms of platelet activation during SARS-CoV-2 infection include specific immunoglobulins found in sera of COVID-19 patients that induce procoagulant platelets *via* FcγRIIa signaling (11, 12), endothelial dysfunction with increased expression of von Willebrand factor (vWF) (56, 57) and stimulation of platelets by proinflammatory markers during the cytokine storm complicating severe cases of COVID-19 (58, 59). Tissue factor (TF) secreted from SARS-CoV-2 infected cells such as epithelium also indirectly activates platelets *via* thrombin-mediated signaling (59, 60). Moreover, thrombopoietin (TPO), which promotes *in vitro* platelet hyperresponsiveness and platelet-leukocyte interaction, is found to be increased in COVID-19 patients (61, 62) Additionally, platelets also secrete cytokines during SARS-CoV-2 infection themselves and consequently contribute to the hyperinflammatory state increasing the risk of CAC (63, 64).

### 2.2 The role of neutrophils and NETs in CAC

Leukocytosis and thus, neutrophilia are common laboratory findings in COVID-19 as the mobilization of immune cells from the bone marrow is one of the earliest responses to combat pathogens (65). The phenotype of neutrophils changes during the infection with SARS-CoV-2. As expected, serum levels of typical markers of neutrophil activation (degranulation and NETosis) such as MPO-DNA complexes or citrullinated histone H3 (Cit-H3) in COVID-19 patients were found to be correlating with disease severity (41, 46). Furthermore, TF increases on neutrophils isolated from patients with severe COVID-19 (66). This suggests how among other pathways primed neutrophils potentially promote or even elicit thrombus formation. As mentioned, NETs are composed of DNA, DNA-associated structures (e.g., histones) and contents of neutrophil granules. Noubouossie et al. reported on the ability of neutrophil DNA to induce thrombin generation (TG) in both platelet-rich and platelet-free plasma although histone-mediated TG appeared to require the presence of platelets (67). For the latter, the toll-like receptors (TLR) 2 and 4 on platelets seem to be of importance in mediating the increase in TG (68).

In a mouse model for SARS-CoV-2 infection, Sung et al. demonstrated the importance of TLR2 and C-type lectin domain family 5 member (CLEC5A) in neutrophils for NETosis and the release of proinflammatory cytokines such as interleukin 6 (IL-6). Interestingly, further *in vitro* experiments with SARS-CoV-2 and mice neutrophils showed accumulation of MPO, Cit-H3 and DNA within the neutrophilic cytoplasm after 5 hours. However, marked NETosis became evident only when incubated with autologous platelets. This suggests that platelet presence could be necessary for further neutrophil activation and NETosis in the case of SARS-CoV-2 infection. Interestingly, in contrast to former evidence with the Dengue virus where NET formation was found to be thread-like, the authors concluded that NETosis elicited by SARS-CoV-2 had a different, more aggregated appearance (69).

Additionally, low-density neutrophils (LDNs) appear to be increasing in number in COVID-19 more than other neutrophil subpopulations (44). These cells - termed CD16^int^ due to their behavior to only intermediately stain with anti-FcγRIII (CD16) – also display an upregulation of genes that are related to NETosis when compared to the CD16^high^ neutrophils. As expected, the authors reported spontaneous *in vitro* formation of NETs in these LDNs (42). Schulte-Schrepping et al. have further elaborated on the myeloid response in severe COVID-19 giving rise to distinct neutrophil precursor subclasses which are characterized by different gene activation signatures including genes involved in NETosis (70). Previously, LDNs have commonly been described in rheumatological diseases as systemic lupus erythematosus or anti-phospholipid syndrome for their proinflammatory effects although consensus on their precise characterization in terms of origin, function and fate has not been reached (71, 72).

As a side note, neutrophils contribute to CAC by releasing a variety of immune mediators causing a cytokine storm and DIC (73). For instance, Kaiser et al. proposed a vicious cycle of IL-8 released from neutrophils in severe COVID-19 which further attracts and activates additional neutrophils (74).

### 2.3 Direct and indirect interplay between platelets and neutrophils in COVID-19

#### 2.3.1 Platelet-neutrophil aggregates as the result of direct interaction

The first quantifiable endpoint of platelet and neutrophil interaction in CAC is represented by complex formation. As mentioned, such platelet-neutrophil aggregates (PNAs) are abundant in SARS-CoV-2 positive patients (37, 41, 45, 75–77). COVID-19 disease severity correlates with blood levels of PNAs (78). Both normal-density neutrophils (NDNs) and LDNs form PNAs although complexes of platelets and CD16^int^ had significantly higher expression of P-selectin (CD62P) and CD40 than PNAs with CD16^high^ neutrophils (42). This could be an additional hint for the hyperactive properties of LDNs and their dominant role in mediating a potential synergy between activated platelets and neutrophils in thrombosis.

**Figure 1:**
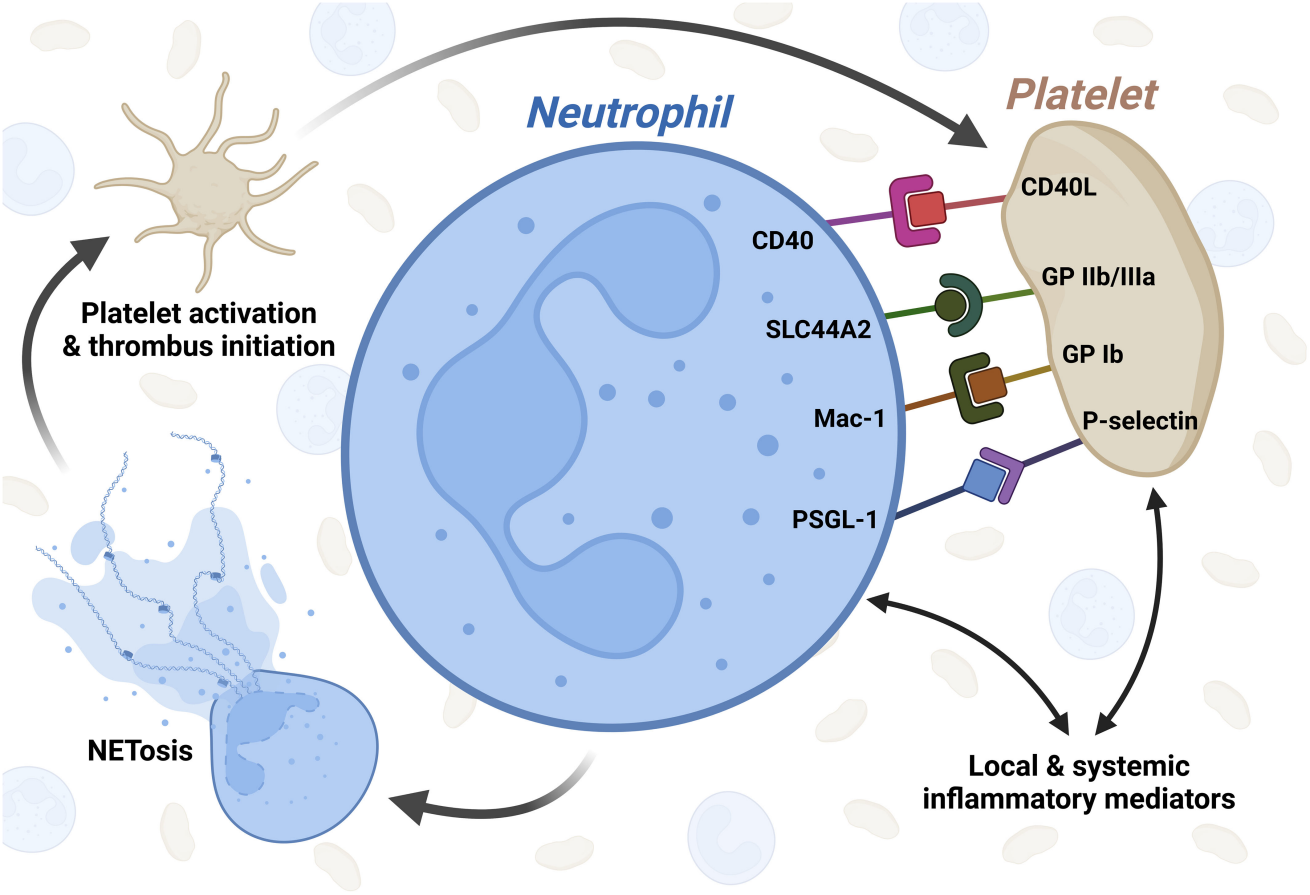
Graphical illustration of platelet-neutrophil interaction in COVID-19. Inflammatory mediators released from immune cells as well as endothelial cells during COVID-19 activate both platelets and neutrophils independently. After activation, several receptors are known to be involved in platelet-neutrophil interaction in COVID-19 which in turn leads to NETosis, subsequent platelet activation and thrombus initiation. CD40L - CD40 ligand; SLC44A2 - solute carrier family 44 member 2; GP IIb/IIIa - glycoprotein IIb/IIIa; Mac-1 - macrophage-1 antigen complex; GP Ib - glycoprotein Ib; PSGL-1 - P-selectin glycoprotein ligand-1. Created with BioRender.com.

In the following, we provide an outline of the most relevant receptors in the process of aggregate formation. **Figure 1** illustrates the direct and indirect aspects of platelet-neutrophil interaction in COVID-19.

##### 2.3.1.1 P-selectin and PSGL-1

Platelet CD62P (P-selectin) and neutrophil P-selectin glycoprotein ligand-1 (PSGL-1, CD162) are a long-known interaction site for platelets and granulocytes (79, 80). Wang et al. demonstrated the major role of PSGL-1 in the coagulopathy associated with systemic inflammation suggesting that CD62P-PSGL-1 coupling also is involved in CAC (81). P-selectin has been identified as the major platelet receptor for monocyte-platelet aggregation in COVID-19 patients (82). Interestingly, platelets *in vitro* activated by SARS-CoV-2 spike protein were shown to cause activation of monocytes *via* CD62P-PSGL-1 coupling (53). Non-specific gene signature analysis of whole blood from severe to critical COVID-19 patients additionally has shown an upregulation of SELPG, the gene encoding PSGL-1 (83).

##### 2.3.1.2 Mac-1

The macrophage-1 antigen (Mac-1) is made up of the two integrins αM (CD11b) and β2 (CD18) and serves several purposes including binding complement and regulation of leukocyte extravasation (84). Despite the name, neutrophils also express Mac-1 and determination of CD11b is considered a typical marker of neutrophil activation beside CD66b (**Table 1**). Previously, it was shown that Mac-1 interacts with platelet glycoprotein Ibα (vWF receptor) in mediating thrombosis (21, 85). Increased expression of both CD11b and CD18 on neutrophils isolated from COVID-19 patients was noted when compared to healthy volunteers suggesting one potential mechanism of platelet activation *via* Mac-1 binding of platelet GP Ib (43, 45). Additionally, the behavior of three different neutrophil subpopulations in COVID-19 patients was investigated by Reyes et al. First, neutrophils isolated by density gradient centrifugation from both the PMN and PBMC layer were separated into NDNs and LDNs. LDNs were further characterized for maturity based on expression of CD10 and CD16. Interestingly, mature LDNs (CD16^+^/CD10^+^) showed high levels of Mac-1 similar to NDNs but formed more complexes with platelets than NDNs. On the other hand, immature LDNs (CD16^-^/CD10^-^) showed lower levels of Mac-1 and appeared to form fewer PNAs (43).

As discussed later, Mac-1 also recognizes several chemokines secreted from platelets including PF4.

##### 2.3.1.3 GP IIb/IIIa and SLC44A2

The platelet glycoprotein IIb/IIIa (integrin α2bβ3, CD41/CD61) is known to interact with the widely distributed choline transporter-like protein 2 (CTL2, SLC44A2) presenting on neutrophils. The importance of SLC44A2 in hemostasis and particularly VTE has already been established in both genetic and animal studies (86–88). Constantinescu-Bercu et al. highlighted the neutrophil SLC44A2 - platelet integrin α2bβ3 axis as an important communication channel of NETosis. Neutrophils were shown to form NETs when infused through GP IIb/IIIa-coated microchannels although simple incubation without flow resulted in a significant decrease in NETosis (89). This implies that formation of NETs also depends on mechanistic effects. From studies on platelet-monocyte interactions, Hottz et al. reported that *in vitro* inhibition of GP IIb/IIIa with abciximab limited the ability of platelets from COVID-19 patients to activate TF expression by monocytes (82). Up to this point there is no data available on SLC44A2 in COVID-19 and how it could potentially impact CAC except for fundamental proteomic data which suggests a significant downregulation of SLC44A2 in neutrophils from severe COVID-19 patients (90).

##### 2.3.1.4 CD40 and CD40L

Apart from many immune responses which are regulated by CD40 and its ligand CD40L (CD154), platelets and neutrophils were also demonstrated to use this pathway (91, 92). Both CD40L expressed on the platelet membrane and soluble CD40L released from platelets (sCD40L) were found to be critical for neutrophil activation in animal models (93, 94). It was also established that CD40L is not uniquely limited to bind CD40 as it also interacted with Mac-1 (95). In general, neutrophil adhesion to platelets was shown to be enhanced by CD40L but this effect was dependent on Mac-1 as its inhibition with anti-CD11b reversed the bonding affinity of neutrophils for platelets (96). Blood from COVID-19 patients had significantly higher concentrations of sCD40L than healthy volunteers (97, 98). However, this was not consistent with the report of Blasi et al. where no significant difference in plasma sCD40L was evident between COVID-19 patients and healthy controls (99). Interestingly, Al-Tamimi et al. showed soluble CD40L levels peaking with moderate disease followed by a decline when disease severity increases (100). This may explain, at least in part, the inconsistencies observed at different time points in the course of COVID-19. *In vitro* stimulation of platelet-rich plasma with the receptor-binding domain of SARS-CoV-2 caused the levels of soluble CD40L to increase (101). This suggests a direct viral effect on platelets causing sCD40L secretion which in turn could induce neutrophil activation. Li et al. showed that spike protein-activated platelets interacted with monocytes using CD40L (53). This clearly highlights the substantiality of platelet presence in fully unfolding the effects of SARS-CoV-2.

On the other hand, the expression of CD40 on LDNs (CD16^int^) correlated with both disease severity and the concentration of D-dimers (42). As described previously, such LDNs are thought to be pro-NETotic. Increased expression of CD40 on LDNs as binding site for platelet surface CD40L and sCD40L released from platelets among other cells (e.g., endothelium) could render these LDNs more susceptible to PNA formation and subsequent platelet-mediated neutrophil activation. For further information on the role of CD40/CD40L in thromboinflammation we refer to the review by Cognasse et al. (102).

#### 2.3.2 Indirect pathways of platelet-neutrophil interaction: inflammatory mediators and microvesicles

Numerous indirect ways of communication between platelets and neutrophils have been reported. Precise dissection of these pathways is challenging and often ambiguous. Most importantly, inflammatory mediators (e.g., cytokines) secreted from both cell types and so-called microvesicles (MVs) are thought to participate in indirect platelet-neutrophil interaction.

Microvesicles are released from cells through membrane budding and usually contain intracellular contents. Platelets are well known to release such extracellular vesicles into circulation in various situations including COVID-19 (63, 64, 103). High levels of platelet-derived MVs expressing TF were found in COVID-19 patients highlighting the thrombotic diathesis of SARS-CoV-2 infection (58, 104). Neutrophils from COVID-19 patients also release MVs which are an important source of TF. Skendros et al. suggested that this platelet-neutrophil-TF axis may be the critical link between immune defense and both plasmatic and cellular hemostasis, leading to CAC (66). Previously, a circular relationship between MVs from neutrophils and platelets has been proposed where direct interaction *via* P-selectin/PSGL-1 coupling initiated platelet-induced arachidonic release from neutrophils. In turn, after uptake into the platelet interior, thromboxane A2 (TxA2) is generated and released causing endothelial activation and subsequently leukocyte rolling and diapedesis (105). Furthermore, TxA2 has been previously reported to play a role in NET formation in the pathogenesis of transfusion related acute lung injury (TRALI) (106). However, whether this applies to NETosis in COVID-19 as well is yet to determine.

The cytokine response to SARS-CoV-2 viremia is complex and sometimes progresses to an hyperinflammatory state with excessive cytokine release (“cytokine storm”). Multiple cell types participate in this process including neutrophils and platelets. As mentioned, platelets in COVID-19 may secrete soluble CD40L but also other non-cytokine mediators such as the positively charged PF4 in COVID-19 (64). PF4 or CXCL4 is known to interact with the neutrophil Mac-1 receptor and also directly with NETs as by their anionic nature. In general, its effects are diverse but include neutrophil chemotaxis, stimulation of NET formation and NET compaction (107–112). The exact role of PF4-mediated platelet-neutrophil interaction in COVID-19 was not investigated further albeit a single study on COVID-19 patients that reported elevated levels of both PF4 and RANTES (Regulated and Normal T cell Expressed and Secreted), a chemokine released from platelets (76). High-mobility group box 1 (HMGB1) also plays a role among the mediators of platelet-neutrophil interplay and high HMGB1 levels were shown to be associated with COVID-19 mortality (113, 114). HMGB1 is a damage-associated molecular pattern (DAMP) protein which can be released from activated or necrotic cells. The function of HMGB1 in thrombosis has only been superficially covered but findings from acute myocardial infarction patients suggested that platelet-derived HMGB1 acts on neutrophils and stimulates the release of NETs. Here, the RAGE receptor (Receptor for Advanced Glycation End products) is of importance (115). An animal study from Vogel et al. has further elaborated on the essential role of HMGB1 in passing prothrombotic signals from platelets to neutrophils (116).

Another example of a relevant mediator in platelet-neutrophil interplay is represented by IL-6 which has already been identified as a main target in counteracting the hyperinflammatory state observed in severe cases of COVID-19 (58, 63). Interestingly, IL-6 blockade in COVID-19 plasma with tocilizumab significantly reduced the high levels of TF+-platelet MVs and PNAs when compared to control plasma (58).

Additionally, neutrophils may release calprotectin or S100A8/A9 upon activation, a protein with the potential to induce procoagulant platelets *via* GP Ibα *in vitro*. COVID-19 patients showed high levels of S100A8/A9 correlating with disease severity (117, 118). Again, differences in upregulation of both S100A8 and S100A9 gene were noted among distinct neutrophil precursor subclasses in severe COVID-19 (70). Additionally, calprotectin deposits have been identified on lung autopsies of COVID-19 deaths (119). The procoagulant effects of neutrophil cathepsin G on the other hand is more certain and direct interaction between this serine protease and platelets is thought to be mediated by protease-activated receptors, PAR-1 and/or PAR-4 (120–123). High levels of cathepsin G are found in COVID-19 but also pneumonia with acute respiratory distress syndrome of different etiologies (124, 125).

### 2.4 Platelets, neutrophils and NETs in VITT

Although little is known about platelet-neutrophil interplay in VITT yet, recent evidence suggests the importance of this relationship in initiating and perpetuating vaccine-induced thrombosis. Direct platelet-neutrophil interaction in the form of PNAs was already found to be upregulated in VITT resulting in higher levels of PNAs as compared to control (126). On a single cellular level, both platelets and neutrophils have been demonstrated to be directly activated *in vitro* by VITT antibodies (126–128). Several case reports have highlighted the presence of NETs within thrombi of VITT patients indicating that NETs are involved in vaccine-induced thrombosis (129, 130). Additionally, increased plasma levels of NET markers (e.g., Cit-H3, MPO-DNA complex) were observed in VITT patients (126, 129). This is in line with recent findings in other prothrombotic conditions such as COVID-19 and HIT. Furthermore, the severity of side effects of the immunization has been correlated to serum histone 3 levels as well (131).

For neutrophils, the proportion of NET-releasing granulocytes was found to be significantly higher in VITT patients compared to control groups (126). Again, LDNs appear to be particularly involved here. NETosis from LDNs in VITT was significantly higher than NET release from NDNs (126, 129). Further research should be directed towards functions and significance of this peculiar neutrophil subpopulation to eventually identify potential pharmaceutical targets in counteracting NET formation in immunothrombosis. Interestingly, Greinacher et al. reported that *in vitro* incubation of isolated neutrophils with VITT serum and PF4 did not lead to NETosis unless platelets were also added to the experiment (128). Thus, it could be concluded that platelets have a crucial role in neutrophil activation and NET formation also in the setting of VITT. Here, microvesicles released from platelets seem to be of importance for the prothrombotic milieu and could help in explaining the cerebral venous tropism of VITT thrombosis (132). However, future efforts are needed to investigate the exact direct and indirect mechanisms of intercellular communication and interplay of neutrophil and platelets in VITT.

Recent studies have focused on neutrophil-activating peptide 2 (NAP2) or CXCL7 released from platelets stimulated with VITT antibodies which in turn activated neutrophils (133–135). Hundelshausen et al. proposed the use of Bruton tyrosine kinase inhibitors (BTKi) in VITT as they were shown to limit platelet P-selectin expression, reduce neutrophil activation and inhibit platelet-neutrophil aggregation (136, 137). Apart from stored VITT sera mainly from spring 2021, future VITT models could rely on chimeric anti-PF4 antibodies mimicking vaccine-induced thrombotic thrombocytopenia such as 1E12 (138).

## 3 Conclusions

In general, both platelets and neutrophils on their own are considered to be major actors in the prothrombotic state seen in COVID-19 and VITT. With our understanding of thromboinflammation still evolving, further efforts should be directed towards dissecting the precise mechanisms of direct and indirect platelet-neutrophil interplay. From our point of view, this relationship should be seen as bidirectional with both types of cells closely interacting and potentiating the thrombotic cascade of initiation, formation and extension of thrombosis. Detailed insights into this interaction and its pathways could be used to design targeted therapies that reduce the occurrence of life-threatening thrombotic complications in COVID-19 and VITT. Additional research on the role of LDNs may help limit the consequences of hyperinflammation associated with COVID-19. *In vitro* thrombosis models, such as microfluidic systems, might be helpful in this regard to understand the role of different cells in the development of thrombosis in patients with COVID-19 as well as VITT.

## Author contributions

JH, GU and TB wrote the manuscript. JH, GU and AS performed the literature review and data collection. JH and JZ designed the Figures. JH, GU, JZ, AS and TB revised the manuscript. All authors contributed to the article and approved the submitted version.
